# Fiber-Type Random Laser Based on a Cylindrical Waveguide with a Disordered Cladding Layer

**DOI:** 10.1038/srep26473

**Published:** 2016-05-25

**Authors:** Wei Li Zhang, Meng Ya Zheng, Rui Ma, Chao Yang Gong, Zhao Ji Yang, Gang Ding Peng, Yun Jiang Rao

**Affiliations:** 1 Key Laboratory of Optical Fiber Sensing & Communications (Education Ministry of China), University of Electronic Science & Technology of China, Chengdu, 611731, China; 2 Photonics & Optical Communications, School of Electrical Engineering & Telecommunications, University of New South Wales, Sydney 2052, NSW, Australia

## Abstract

This letter reports a fiber-type random laser (RL) which is made from a capillary coated with a disordered layer at its internal surface and filled with a gain (laser dye) solution in the core region. This fiber-type optical structure, with the disordered layer providing randomly scattered light into the gain region and the cylindrical waveguide providing confinement of light, assists the formation of random lasing modes and enables a flexible and efficient way of making random lasers. We found that the RL is sensitive to laser dye concentration in the core region and there exists a fine exponential relationship between the lasing intensity and particle concentration in the gain solution. The proposed structure could be a fine platform of realizing random lasing and random lasing based sensing.

Different from traditional lasers, random lasers (RLs) do not need a resonance cavity with high-quality reflection mirrors. Their working principle is based on amplified multiple scattering in disordered systems^1^,^2^,^3^. Thanks to relatively strong scattering, feedback loops of light might be formed, corresponding to special cavities of RLs. In this case, random lasing actions will emerge when gain of the laser cavity is larger than its loss. Since the pioneer works in 1990s, RLs have been realized with a wide range of materials, such as semiconductor, polymer, liquid crystal, biological material/tissue, and optical fiber, covering radiation wavelengths from UV to Mid-infrared^2^,^3^,^4^,^5^,^6^,^7^.

To enhance the emission efficiency, tailor the output spectrum, or control the emission directions of random lasing, gain materials and scatters of RLs were embedded into optical waveguide structures^3^,^8^,^9^,^10^,^11^,^12^,^13^,^14^, in which light amplification and scattering are confined and mediated by the waveguide, giving birth to partially regulated and still randomly formed positive feedback loops that support random lasing. Generally speaking, there are two ways of generating amplified multiple scattering to form positive feedback loops in RLs, i.e., 1) doping randomly distributed scatters into the gain material and 2) forming a random structure adjacent to the bulk gain material.

For the first type of RLs, the gain material and the scatter are mixed together, and they have been reported in semiconductor waveguides, filled-core or solid-core optical fibers, etc.^8^,^9^,^10^,^11^,^12^,^13^,^14^. For the second type of RLs, the gain material and the scatter are in separate regions and this facilitates controlling of light amplification and scattering process separately. The second type has been reported in photonic crystal membrane waveguides with engineered disorders, a polydimethylsiloxane waveguide with gain solution in a rough microfluidic channel, and dye solution between disordered structures^3^,^15^,^16^.

In this paper, a novel RL of the second type is proposed, making from a fiber-type cylindrical waveguide that is formed by coating a disordered dielectric film in the internal surface of a glass capillary, and filling the capillary with dye solution. Compared with previous work, the proposed RL is easier to be fabricated because its scattering and gain can be simply and separately controlled by changing the concentrations of scatter and gain materials. In addition, the proposed fiber-type waveguide with disordered cladding also provides guide/confinement of light that assists formation of random lasing modes, and enables random lasing with narrow linewidth and wide emission wavelength range. Taking advantage of the sensitive characteristics of RL to small particles^17^, a method of using random lasing to detect the concentration of particles in the dye solution is proposed for the first time to the best of our knowledge.

## Experimental Setup

**Figure 1:**
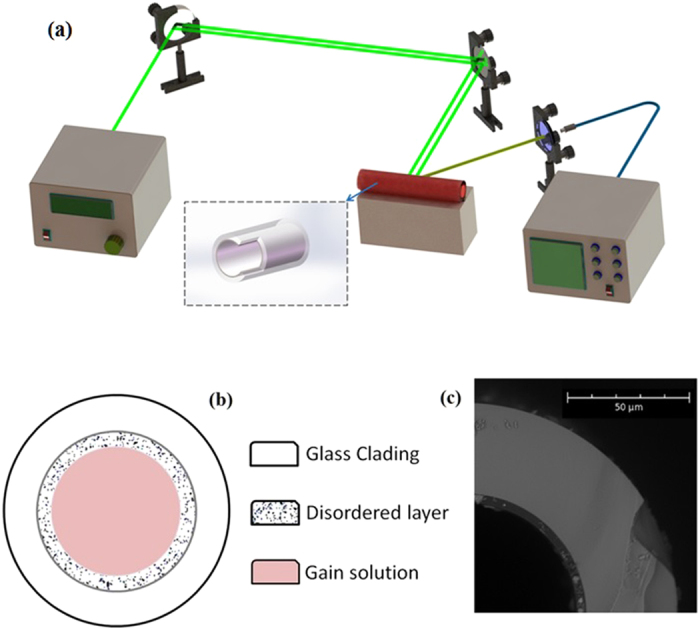
Schematic diagram of the experimental setup and the RL structure. (**a**) Experimental setup, (**b**) RL structure, (**c**) Scanning electron microscope image of the cross-section of the cylindrical waveguide with coating.

The experimental setup of this work is given in Fig. 1(a). A Nd:YAG laser radiates 532 nm pulse with 10 ns pulse width and 20 Hz repetition rate is used to pump a sample of the RL. To guide the pump light, a transparent glass plate and a glass mirror is used as reflectors. The transparent glass plate reflects the pump light at both its front and back surfaces (i.e., two interfaces between the plate and air) and separates the pump light to two spots (each of the spot has a diameter of ~3 mm). Both the spots are reflected by the glass mirror and shine on the sample at slightly different regions so that the pumped area is about doubled and the lasing threshold is reduced (Here we use this simple method to increase the pump area without using additional components to tune the waist of the pump light). Emission of the sample is collected, through a collimator, by a bunch of fibers that are connected with an optical spectrum analyzer (OSA), and the acquisition time of the OSA is 1 *s* (thus, the spectra obtained in the successive studies are average outputs other than statistical ones^18^,^19^).

The sample is prepared in two steps. Firstly, UV-Cured adhesive mixed with TiO_2_ nano particles (mass ratio of the TiO_2_ particles and the adhesive is 1:4) is injected into a capillary (with outer and inner diameters of 170 and 100 μm, respectively). The filled capillary is then inflated with steady flow of air for 3 minutes, coating a disordered thin film (i.e., about 3 μm thick) on the inner surface of the capillary, as shown by Fig. 1(b,c). To solidify and stabilize the coating layer, an ultraviolet light is used to irradiate the capillary. Secondly, Rhodamine B solution (the concentration of Rhodamine B in deionized water is 1 mol/ml) is filled into the coated capillary as the gain material. The coating layer has higher refractive index (~1.5), and the filled core has lower refractive index (~1.39). The coating layer with randomly distributed TiO_2_ particles can scatter light back into the gain region. Thus, light localization and positive feedback loops of light might form randomly close to interface of the gain region and the coating layer^1^, giving birth to random lasing actions.

### Numerical Analysis

**Figure 2:**
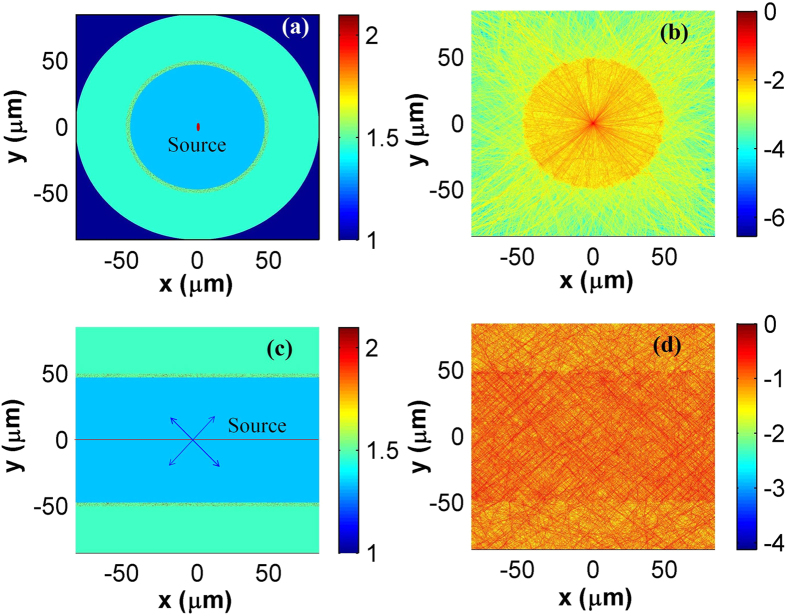
Refractive and light intensity distribution at 595 nm. (**a**–**d**) are the refractive index and normalized light intensity (in logarithmic scale) distribution of the vertical (parallel) section respectively.

Light distribution in the proposed structure is analyzed through Finite Difference Time Domain (FDTD) method^20^,^21^. To simplify the simulation, sections vertical to and parallel to the axial direction of capillary are simulated. Figure 2(a) shows the refractive index distribution of the vertical section. To mimic light emission in the radial direction, an electric dipole light source is added at the center of the vertical section. From Fig. 2(b) we can find that the emitted light is multiply scattered and partially confined in the core by the coated layer. Figure 2(c) shows the refractive index distribution of the parallel section, and four plan wave sources (i.e. with radiation direction of 45, 135, 225 and 315 degrees respectively) set at the axis of the parallel section are used to mimic light emission in both the radial and the axial directions. Similarly, Fig. 2(d) indicates that, the emitted light will be multiply scattered and partially confined in the core. Thus, the proposed structure performs like a fiber that assists formation of random laser modes in the core region, and random lasing might be supported if gain mechanism is provided.

## Experimental Results

**Figure 3:**
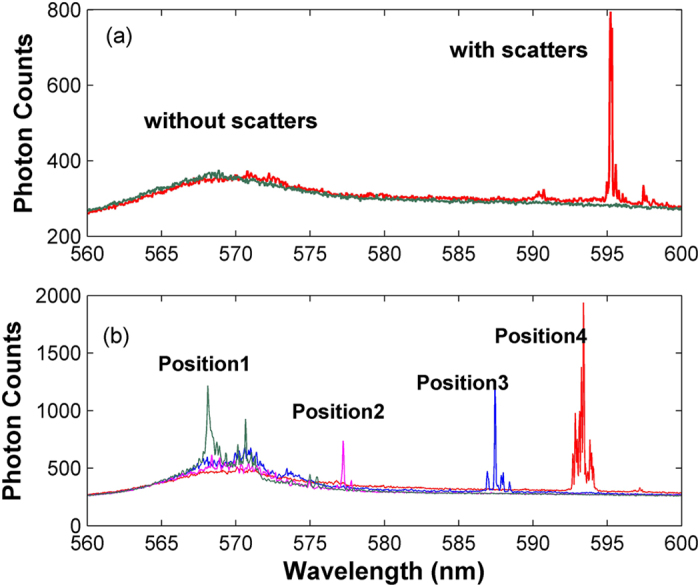
Output spectra of the RL. (**a**) Output spectra of the samples with and without scatters in the coating layer, (**b**) Output spectra of the RL for different pump positions. The Rhodamine B concentration is 1 mol/l, and the pump intensity is 0.645 W/cm^2^.

To verify our assumption, Fig. 3(a) compares the emission spectra between two different samples under same pump conditions. When there are no TiO_2_ particles doped in the coating layer (Sample 1), the output spectrum is wideband amplified spontaneous emission (ASE). Besides, there is no whisper gallery mode (WGM) observed in the spectrum, indicating that the coated layer is neither fine enough to support lasing of WGMs nor nonuniform enough to provide strong scattering for random lasing^17^. When the TiO_2_ particles are doped in the coating layer (Sample 2), strength of light scattering is enhanced greatly, and random lasing with narrow linewidth is obtained.

We also repeated the experiment of Fig. 3(a) by irradiating the pump at different axial positions of the samples, which is realized by changing the relative position of the samples, while the pump and the collection positions keep unchanged. For Sample 1, no lasing action is observed, and the output is still ASE, which further verifies that only the waveguide effect is not enough to support laser emission. For Sample 2, lasing at different wavelengths has been observed when changing the pumping positions (e.g. from positions 1–4 as shown in Fig. 3(b)). Such pump-position-depended output is a typical characteristic of RLs, which corresponds to the formation of different cavity modes through multiple scattering at different part of the sample. In our case, the RL wavelength can be tuned in a relative large bandwidth, i.e., from ~570 to ~600 nm, without changing parameters of its gain material or scatters. This is because, 1) large length to volume ratio of the RL (i.e., quasi-one dimensional structure) increases nonuniform distribution of light localization along its axial direction, and 2) the waveguide effect of the proposed structure assists/mediates the formation of localized modes apart from random scattering, so laser modes within wider wavelength region might be formed with positive net gain. For example, random lasing is observed not only at the maximum of the gain profile, because lasing wavelength is determined not only by the gain maximum of the dye solution but also by the loss of the randomly formed laser mode (i.e., the filtering effect of the waveguide together with the gain mechanism determines the wavelength of maximum net gain). In our experiment, we also tested the cases when either one of the two pump spots is blocked, and no random lasing is observed. This reflects that the random lasing mode extends in the axial direction of the sample, and the waveguide certainly takes effect.

**Figure 4:**
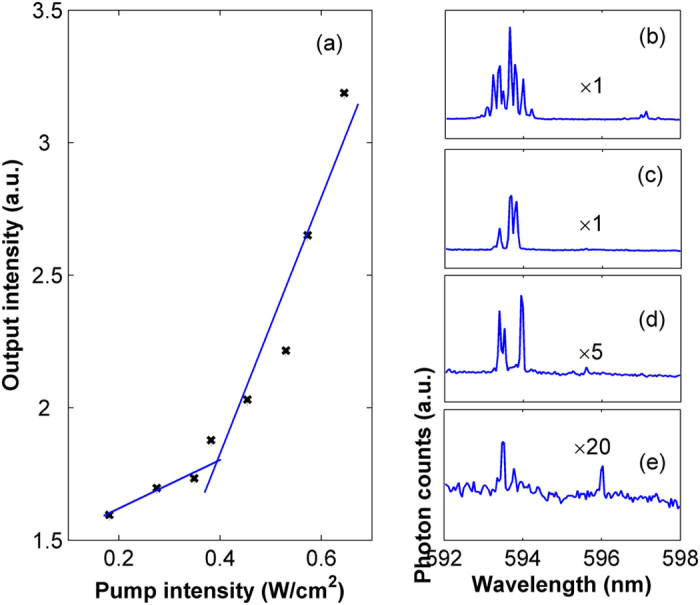
Output characteristics of the RL. (**a**) Output power versus pump intensity, (**b–e**) Output spectra for pump power of 0.645 W/cm^2^, 0.529 W/cm^2^, 0.382 W/cm^2^ and 0.275 W/cm^2^ respectively.

Taking the random lasing action when pump is at position 4 as an example, output characteristic of the RL is given in Fig. 4. It is observed that the output intensity increases with the pump intensity, and clearly exhibit a threshold behavior when the pump intensity is ~0.36 W/cm^2^, as seen in Fig. 4(a). The laser spectra corresponding to different pump intensities are shown in Fig. 4(b–e). It is observed that lasing peaks around 594 nm start to emerge when pump intensity is larger than the threshold. With increase of the pump intensity, more and more lasing peaks emerge, which is also one typical phenomenon of random lasing action.

In our experiment, the gain solution is filled into the coated capillary by a micropump through connection of a plastic tube. It is very convenience to change the gain solution using the same coated capillary. Thus, an open random microcavity is formed wherein the gain medium is replaceable, offering a promising way for optical microfluidic applications based on random lasing.

**Figure 5:**
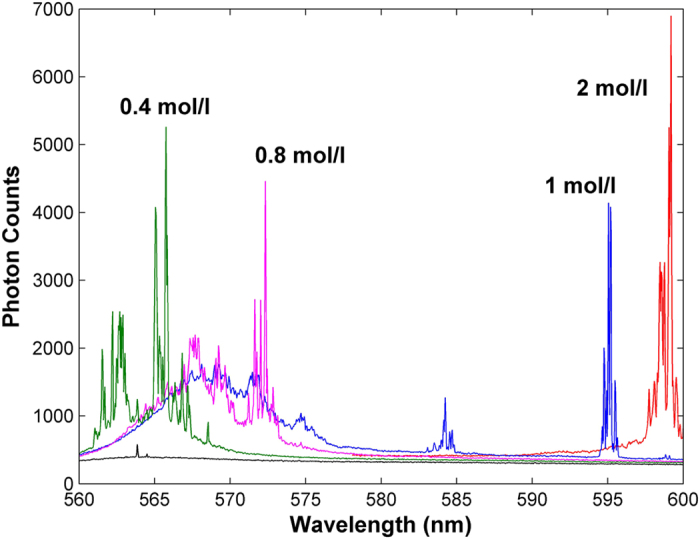
Output spectra of the RL for different concentration of gain material. The pump intensity is 0.645 W/cm^2^.

In the following study, we investigate emission wavelength of the RL by changing concentration of the gain solution. It is observed (Fig. 5) that random lasing peaks change from 560 to 600 nm when concentration of the gain solution changes from 0.4 to 2 mol/l. This is due to wavelength blue-shift of the gain maximum^22^.

**Figure 6:**
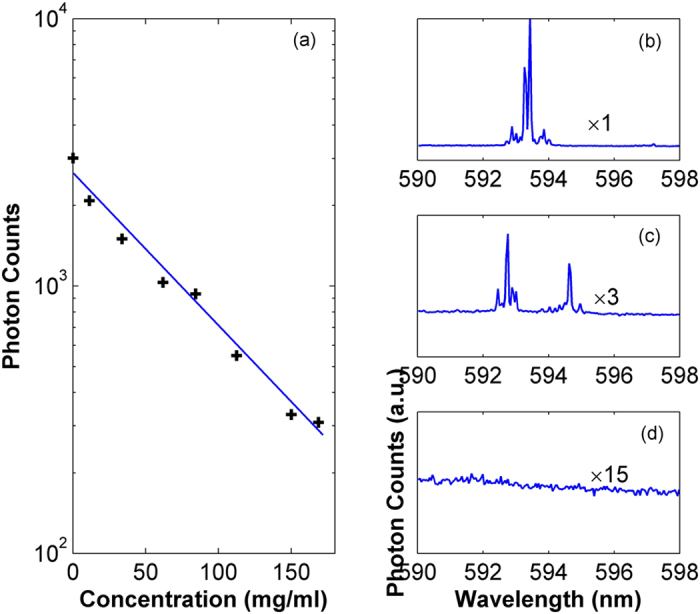
Output characteristics of the RL with polyethylene particle in the gain solution. (**a**) Maximum photon number of emission versus concentration of the polyethylene particles. (**b–d**) Output spectra for polyethylene particles concentration of 0, 50, and 150 mg/ml, respectively.

We also mix polyethylene particles of different concentrations into the gain solution. It is observed that the maximum photon counts (i.e., average value of 10 measurements) decreases exponentially with increase of particle concentration, as seen in Fig. 6(a). Spectra of the RL indicate that the emission wavelengths also vary with particle concentration in the gain solution, as shown by Fig. 6(b–d). This is because that the particles added to the gain solution will scatter light and change trajectories of light propagation. As a result, the chance to form laser cavities with positive net gains is reduced, and different laser cavities might be formed, giving birth to different lasing modes with reduced output intensity.

## Discussion and Conclusion

It is worth mentioning that the proposed method may be easily applied for RLs using other gain materials/solutions, by replacing the core solutions and the appropriate pump source. Hence it provides a simple and flexible platform using microfluid techniques for developing RLs of different gain materials for “on-chip” sensing applications. The proposed structure can also be incorporated in traditional optical fibers (e.g. adding a random cladding layer to the core), which can provide scattering much stronger than merely Rayleigh scattering in the fiber core^10^,^11^,^12^,^13^, and could be an efficient way to reduce threshold and cavity length of traditional random distributed feedback lasers. It is worth of noting that the above simulation results indicate that the proposed structure can perform like a waveguide to trap most of the light in the core, however, part of the light will be scattered out of the core in random directions. When using this structure to make real fibers, stronger scattering is helpful to provide light feedback, but this will also increase loss. Thus, balance between useful and harmful scattering should be considered by selecting dimension and refractive of each layer of the fiber.

In conclusion, a fiber-type random laser based on a cylindrical waveguide with a disordered coating layer is proposed and demonstrated. The waveguide effect together with the amplified light scattering of the proposed structure provide efficient mechanism to form RL modes, and contribute to random lasing with narrow linewidth and wide wavelength tunability. The random lasing intensity decreases exponentially with increase of particle concentration in the core, providing a potential way of small particle sensing using RLs.

## Additional Information

**How to cite this article**: Zhang, W. L. *et al*. Fiber-Type Random Laser Based on a Cylindrical Waveguide with a Disordered Cladding Layer. *Sci. Rep.* **6**, 26473; doi: 10.1038/srep26473 (2016).
